# Genetic characterization of four strains porcine circovirus-like viruses in pigs with diarrhea in Hunan Province of China

**DOI:** 10.3389/fmicb.2023.1126707

**Published:** 2023-03-01

**Authors:** Chihai Ji, Meng Zeng, Yingfang Wei, Xiaocheng Lv, Yuan Sun, Jingyun Ma

**Affiliations:** ^1^Guangdong Provincial Key Lab of Agro-Animal Genomics and Molecular Breeding, College of Animal Science, South China Agricultural University, Guangzhou, China; ^2^Guangdong Laboratory for Lingnan Modern Agriculture, College of Animal Science, South China Agricultural University, Guangzhou, China; ^3^Key Laboratory of Animal Health Aquaculture and Environmental Control, College of Animal Science, South China Agricultural University, Guangzhou, China; ^4^College of Veterinary Medicine, South China Agricultural University, Guangzhou, China

**Keywords:** CRESS DNA virus, Po-Circo-like virus, Rep protein, phylogenetic analysis, epidemiologic

## Abstract

In this study, we detected a circular replication-associated protein (Rep)-encoding single-stranded (CRESS) DNA virus [named Po-Circo-like (PCL) virus] in intestinal tissue and fecal samples of pigs. PCL virus contains a single-stranded DNA genome, and ORF1 encodes the Rep and not the typical capsid protein encoded in PCV. The Rep protein may be responsible for viral genome replication. In addition, PCL virus may be one of the pathogens causing diarrhea symptoms in pigs. We identified four strains of PCL virus in two different pig farms with severe diarrhea outbreaks in Hunan Province, China. The strains in this study share 85.7–99.7% nucleic acid identity and 84.7–100% amino acid identity with Rep of the reference strains. A multiple sequence alignment of these PCL viruses and Bo-Circo-like CH showed a identity of 93.2% for the Rep protein, and the nucleotide identity was 86.7–89.3%. Moreover, Bo-Circo-like CH and HN75, HN39-01, HN39-02 had similar stem-loop sequences. In conclusion, the present study is the first detailed report of the PCL virus in Hunan provinces, which is a potential new virus in pigs that might be involved in cross-species transmission. Further investigation is needed to determine the pathogenesis of this virus and its epidemiologic impact.

## 1. Introduction

The circular, Rep-encoding Single-stranded (ss) DNA (CRESS-DNA) viruses are wide spread in various habitats and show high diversity (Shulman and Davidson, 2017; Kazlauskas et al., 2019). In recent years, an increasing number of CRESS viruses have been reported with the help of the high-throughput sequencing technologies. PCL virus (porcine circovirus-like virus) is a new type of CRESS virus that belongs to the proposed Kirkoviridae family (Liu et al., 2021). PCL virus was first discovered in pig stool by American scholar Shan in 2011 and was reported in China in 2014 (Shan et al., 2011; Zhang et al., 2014; Sun et al., 2021). Existing epidemiological investigations of PCL virus have shown that PCL virus mainly infects piglets, causing symptoms such as diarrhea, hemorrhagic enteritis. Yang et al. (2021) detected PCL virus in a dead sow with porcine epidemic diarrhea virus (PEDV) in Anhui Province. In addition to PEDV, pigs infected with PCL virus are also co-infected with viruses such as porcine circovirus type 2 (PCV-2), porcine parvovirus (PPV; Sun et al., 2021; Yang et al., 2021; Zeng et al., 2022). In this study, PCL virus was detected for the first time and in pregnant sows in Hunan Province, which greatly enriched the epidemiological information of PCL virus. It is noteworthy that pigs infected with PCL virus had diarrhea symptoms, but some sows showed abortion. Further investigation is needed to determine the relationship between the virus infection, diarrhea and abortion.

*Circoviridae* is divided into two genera of *Circovirus* and *Cyclovirus*, and can infect a variety of animals (Li et al., 2011; Phan et al., 2014). In general, circoviruses are small, single-stranded, linear, non-enveloped DNA viruses with a genome approximately 1.7–2.1 kb in length (Breitbart et al., 2017). Their genomes contain two major open reading frames (ORFs) that encode the replication-associated protein (Rep) and the sole structural protein (capsid protein, Cap). PCL virus has a similar genome to PCVs, as a circular genome and a stem-loop. However, all the amino acid sequences of Rep proteins of the viruses belonging to proposed family Kirkoviridae indicate significant genetic distance with those of the viruses within family *Circoviridae* (Shan et al., 2011; Guo et al., 2018; Sun et al., 2021). In fact, PCL viruses have little nucleotide identity with PCV. In recent years, more PCL virus have been reported one after another. Nowadays PCL virus has three predicted types of the stem loop:GGGCAATTCTGCCC, GGGCAAGTCTGCCC, and GGGCAAATCTGCCC. Surprisingly, there was a substitution in the stem loop of the PCL virus (Cheung, 2007; Liu et al., 2021; Sun et al., 2021). In 2018, Guo et al. (2018) discovered a novel CRESS DNA virus from a calf with severe hemorrhagic enteritis. However the replication proteins (Rep) of Bo-Circo-like virus CH showed extremely high identity with Po-Circo-like virus (Liu et al., 2021). This finding indicated that the PCL virus is different from PCV and is similar to Bo-Circo-like. Rep proteins have been proposed for use as the basis for megataxonomic classification of CRESS DNA viruses (Liu et al., 2014; Koonin et al., 2020). Fewer amino acid mutations were observed on the Rep protein of these viruses (Liu et al., 2021). The current study, phylogenetic analysis also based on the replication proteins (Rep), divides PCL virus into two subpopulations PCLa and PCLb (Cheung, 2007). In the past few years, research has focused on the genetic characteristics and evolution of PCL viruses. No cell lines have been identified for isolation of the virus. Unfortunately, we attempted to use PK15 cell lines to isolate the virus, which was not successful. So research on the pathogenesis of PCL virus is currently stagnant. This study was the first to report the detection of the PCL virus in Hunan provinces of China. However, its clinical significance, epidemiology and disease prevention deserve further in-depth study.

## 2. Materials and methods

### 2.1. Sample collection

Farm A: An outbreak of hemorrhagic enteritis, diarrhea, anorexia and vomiting of piglets and diarrhea of sows of unknown cause occurred in a large-scale pig farm in Chenzhou, Hunan Province, on 10 February 2022. Thirty fecal swabs were collected. Farm B: On 12 February 2022, a large-scale pig farm in Chenzhou, Hunan Province showed similar symptoms to farm A, and six small intestine samples of piglets were collected.

### 2.2. PCR assays

Clinical samples mixed with phosphate-buffered saline (PBS) and repeatedly freeze-thawed three times. The viral DNA/RNA is then extracted using RaPure Viral RNA/DNA Kit (Magen, R4410-3, China). Polymerase chain reaction (PCR) assays for the detection of several common pig viruses w-ere used including porcine epidemic diarrhea virus (PEDV), Transmissible gastroenteritis virus of swine (TGEV), Swine acute diarrhea syndrome coronavirus (SADS-CoV) and porcine deltacoronavirus (PDCoV). Five primer pairs were designed based on the reference sequence of the PCL virus 21 and 22 strain determined in the United States and the PCL virus detected in China. The primers are described in Table 1.

**Table 1:** List of primer sequences used in this study.

Primer name	Primer sequence (5′–3′)	Size (bp)
PCLV-3818F	TAGGGATTTCCGCTTGGATCAAGTACT	1,343
PCL-1202R	TATTACCTTTAGCGGAATCAAAATCGGAC	
PCL-982F	ATGCCTGGCACCTTAGACCCCTTTA	944
PCL-1905R	AATGAACTGACCACTCATGAA	
PCL-1859F	GTTAACCGCAATTGAATTTTGA	1,207
PCL-3045R	GCAGGCGTATGGCCGGGTGAT	
PCLV-2901F	TTGGCCTTTACGTGTATAATACCT	1,201
PCLV-149R	TTCCTCTTCGTAAGTAAAGTTGTT	
PCLV-3378F	GTCTCAGGACTTCGATGTATTCGACCCTT	550
PCLV-3904R	TCTCAGGACTTTGATGTATTCGAC	
TGEV-F	GTATAAAACCTCCTGGCTGT	780
TGEV-R	GCCATTGATTTATGGAGACA	
PEDV-F	GTCTTACATGCGAATTGACC	526
PEDV-R	CAACCTTATAGCCCTCTACA	
PDCoVF	ATCCTCCAAGGAGGCTATGC	493
PDCoVR	GCGAATTCTGGATCGTTGTT	
SADS-CoV F	CCCTATGTGTGTAACACATCTGGT	367
SADS-CoV R	ACTTCCCATTGCAACAGTAGTTCTT	

### 2.3. PCL virus sequencing of the full-length genome

PCR products of PCL viruses positive samples were amplified and purified. We cloned it into the pMD 19-T vector (TaKaRa Bio Inc.) and then transformed it into DH5α competent cells. The positive clones were screened by PCR and sent to Sangon Biotech (Shanghai) Co., Ltd. for sequencing.

### 2.4. Sequence alignment, phylogenetic analysis

The complete gene sequences of PCL viruses obtained in this study have been uploaded to Genbank with the accession numbers (HN14: GenBank No. OP302750; HN75: GenBank No. OP302751; HN39-01: GenBank No. OP302752; and HN39-02: GenBank No. OP302753). BLAST analysis^1^ was done on all obtained sequences to check for homologous sequences in GenBank. The genome lines were assemble using Lasergene and SnapGene software (version 4.2.4; from Insightful Science; available at snapgene.com). Subsequently, all gene sequences arrangements were further aligned with MegAlign (Lasergene) using the Clustal W alignment method. A phylogenetic tree was built using the maximum likelihood method with 1,000 bootstrap replicates in MEGA 7.0 software. The reference strains of this study are shown in Supplementary Table 2, including GenBank accession number and strains name.

## 3. Results

### 3.1. Detection of PCL virus in clinical specimens

Since mixed infection of pathogens is common in clinically infected pigs, we used polymerase chain reaction (PCR) to detect PEDV, TGEV, SADS-CoV, PDCoV, PCL virus. The primers are described in Table 1. A total of 36 samples were collected from two different pig farms in Chenzhou City, Hunan Province, China. Of the 30 samples collected from farm A, three were PCLV positive, with a positive rate of 10% (3/30). Of the six samples collected from B farm, one was PCLV positive, with a positive rate of 16.7% (1/6). The total positive rate of PCL virus was 11.1% (4/36). We numbered the positive samples, numbers 1–3 from Farm A and 4 from farm B. Sample 1 (HN14, Accession No. OP302750), the mixed infection of PCL virus with SADS-CoV and PEDV was also detected. The results showed that there was mixed infection in farm A. Samples 2–4 (HN75, HN39-01, and HN39-02; Accession No. OP302751–OP302753) detected only PCL virus.

### 3.2. Sequence, phylogenetic analysis

**Figure 1:**
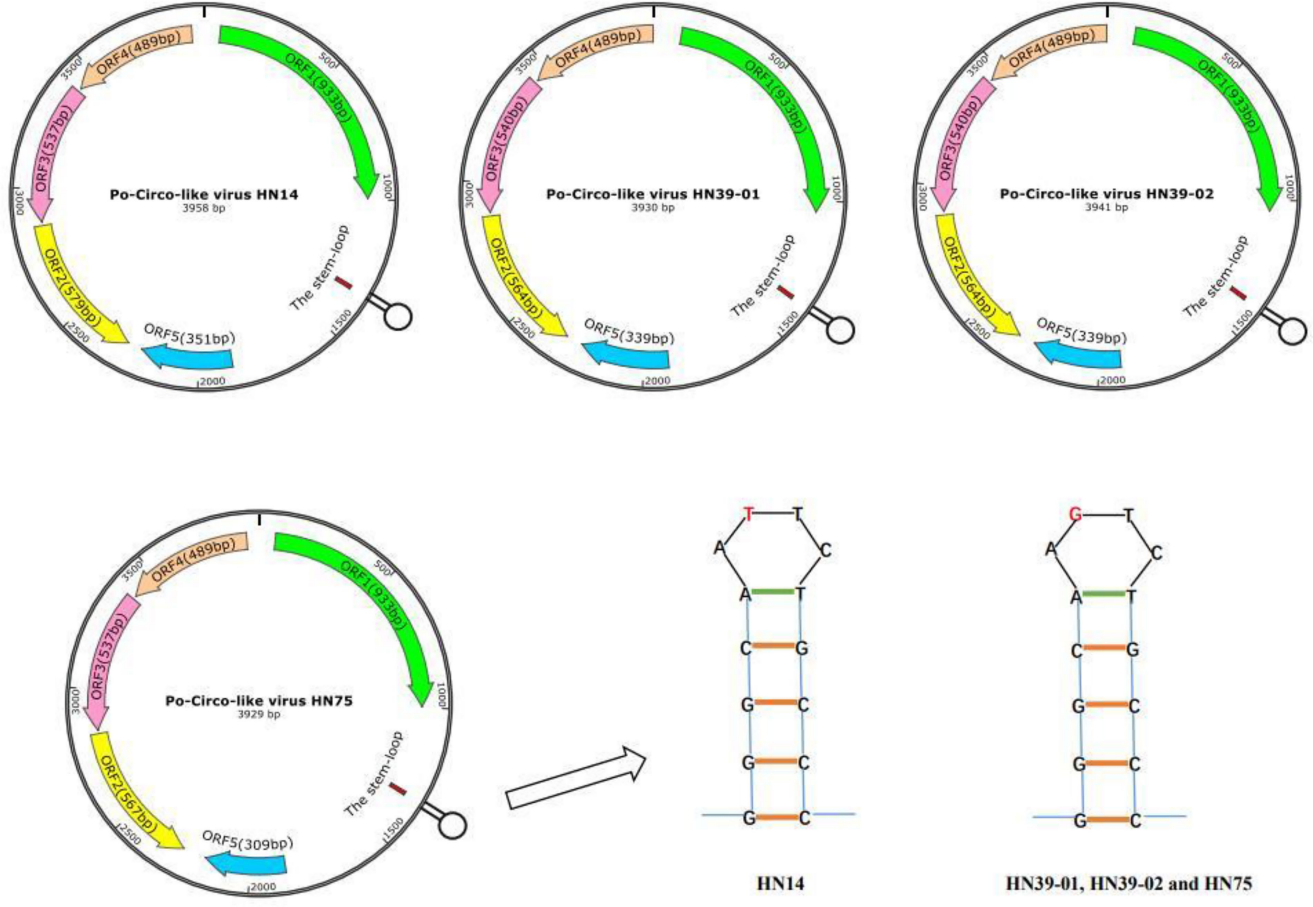
Predicted genome organization of the Po-Circo-like virus and the stem loop of the Po-Circo-like virus.

Subsequently, the full genomes of four strains were sequenced, and their gene characteristics were further analyzed (Figure 1). First, SnapGene software (version 4.2.4; from Insightful Science; available at snapgene.com) was used to assemble the genome sequences together and draw the complete gene map. The NCBI Open Reading Frame (ORF) Finder^2^ was used to identify the putative main ORFs of PCL virus. The stem-loop structure of PCL virus was verified using the Mfold web server (Zuker, 2003; Hao et al., 2021).^3^ The genomes of these strains were all circular, with a Rep protein and a stem loop of the same length (Figure 1). Five potential ORFs, each with at least 100 amino acids, were identified. Of the revealed ORFs, the largest ORF was named ORF1, and it enco-des a replication-associated protein (Rep). PCVs have a stem-loop structure (Cheung, 2007), while PCL viruses also have a stem-loop containing 14 nucleotides (Sun et al., 2021). Unlike PCVs, PCL viruses do not express the Cap protein. Stem loop of four strains PCL virus includes GGGCAATTCTGCCC and GGGCAAGTCTGCCC (Figure 1). The effect of stem-loop structure on virus needs to be further studied.

**Figure 2:**
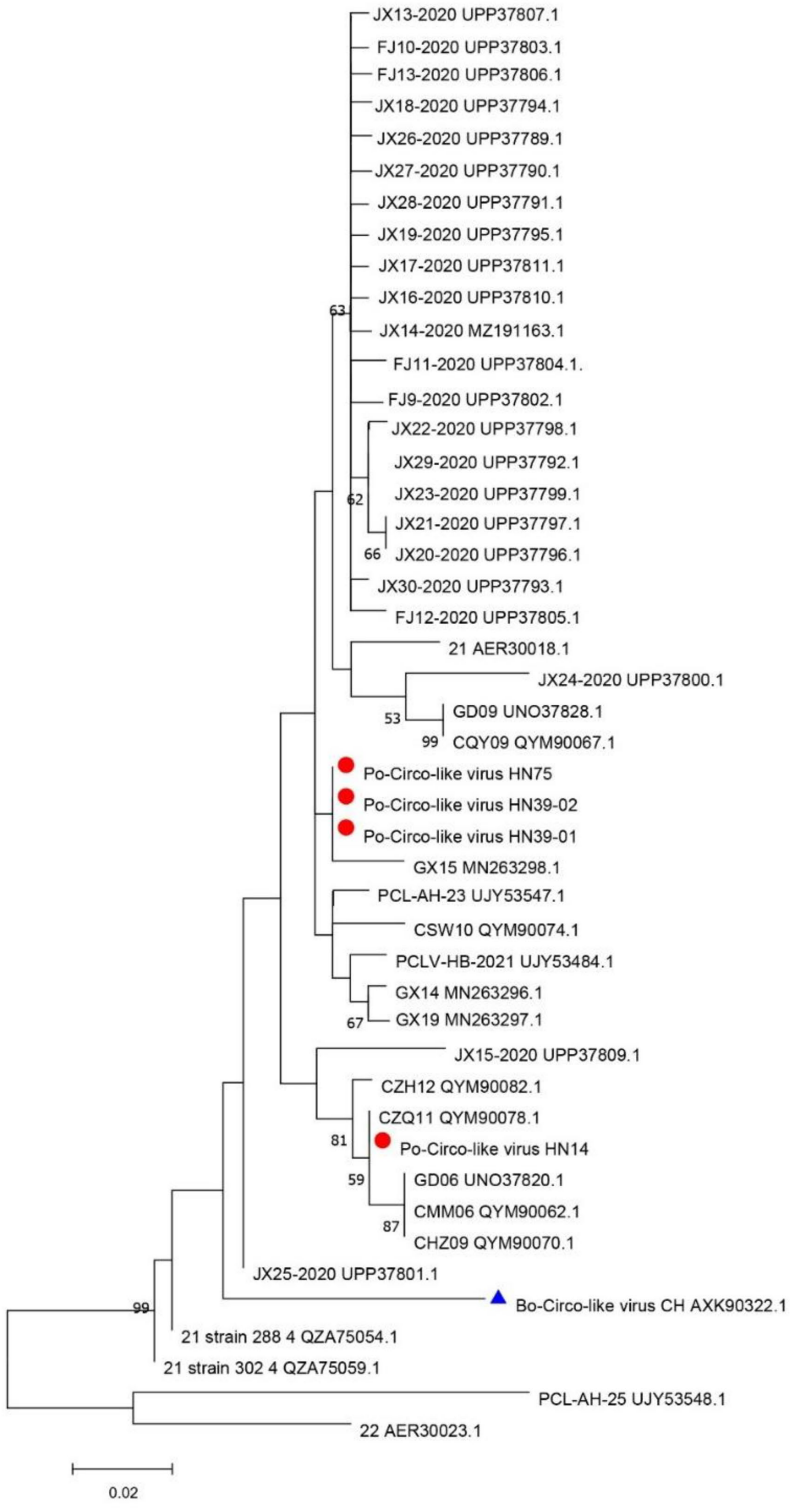
Phylogenetic trees based on the rep gene sequences of the Po-Circo-like virus and the Bo-Circo-like virus. The evolutionary history was inferred by using the Maximum Likelihood method based on the JTT matrix-based model, and 1,000 bootstrap replicates in MEGA 7.0 software. The percentage of trees in which the associated taxa clustered together is shown next to the branches. Red circles indicate the strains detected in this study, and blue triangle indicate the strains that were derived from cattle.

The DNASTAR package (DNASTAR, Madison, WI, United States) was used for sequence alignment and identity analysis. The strains in this study share 85.7–99.7% nucleic acid identity and 84.7–100% amino acid identity with Rep of the reference strains (Supplementary Table 2). A multiple sequence alignment of these PCL viruses and Bo-Circo-like CH showed a identity of 93.2% for the Rep protein, and the nucleotide identity was 86.7–89.3% (Supplementary Table 2). In this study, HN14 strain had similar stem-loop sequence with GX14 and GX15. Moreover, HN75, HN39-01, HN39-02 and Bo-Circo-like CH, GX19 had similar stem-loop sequences. Some studies have shown that Bo-circo-like CH and PCL viruses have high similarity, which is consistent with our results (Liu et al., 2021; Sun et al., 2021). One hypothesis that might explain this finding is that the Bo-Circo-like virus CH may be a PCL virus that infects calves. In summary, the results indicated that it is possible that the PCL virus is transmitted to non-porcine hosts. The ORF1 sequences of the Chinese and American endemic strains were downloaded from GenBank. All gene sequence were further aligned with MegAlign (Lasergene) using Clustal W alignment. MEGA 11 software was used to construct the phylogenetic tree with 1,000 bootstrap repeats using the maximum likelihood method (Figure 2). The results showed that PCL virus, Bo-Circo-like virus, and other viruses were clustered in one clade. In the present study, these novel CRESS DNA viruses were classified into the new family Kirkoviridae (Li et al., 2010; Liu et al., 2021).

## 4. Discussion

**Figure 3:**
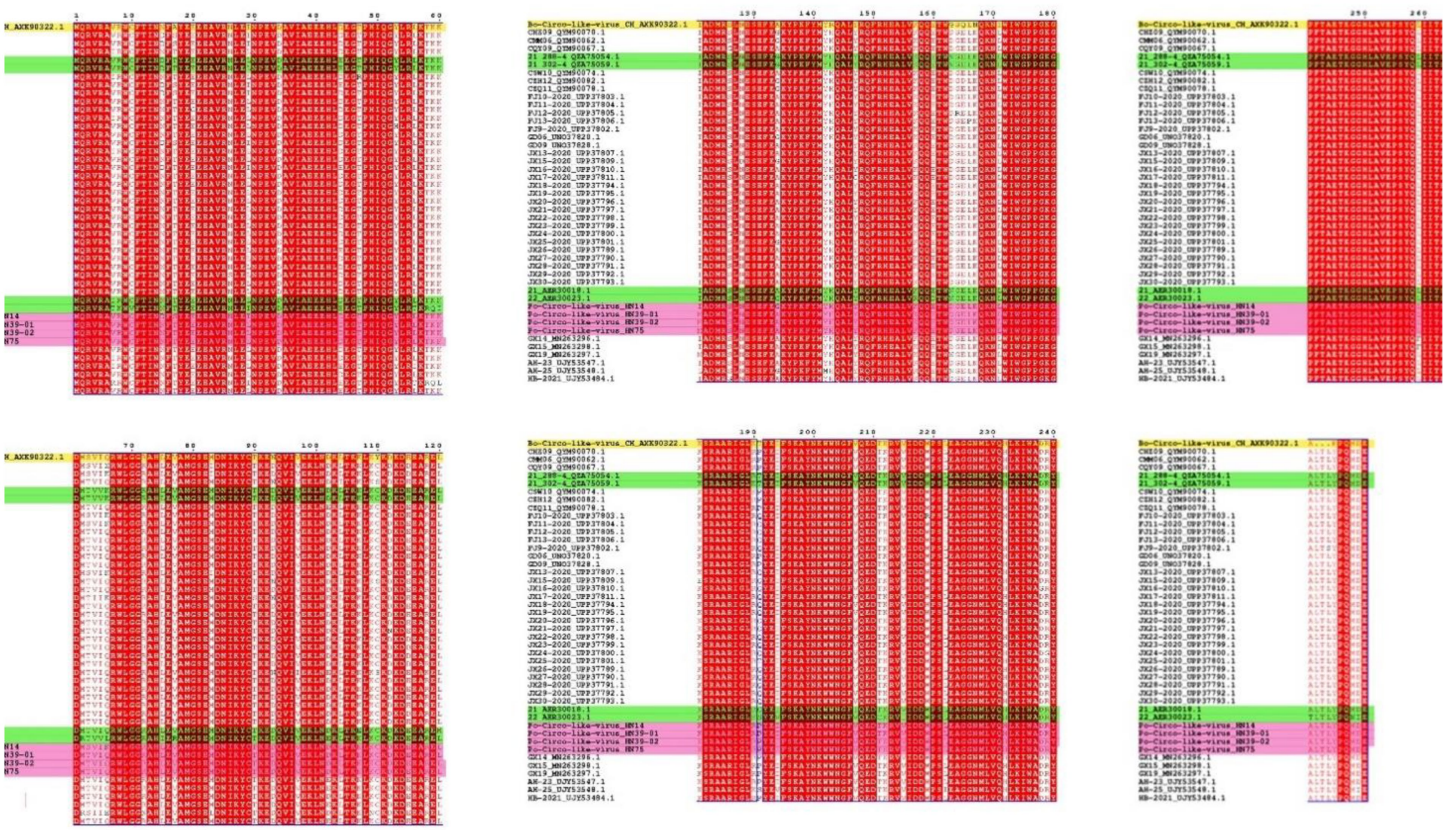
Amino acid comparison and analysis on Rep protein. The strains in this study are marked in pink. Yellow indicates the Bo-Circo-like virus, and unlabeled strains indicate of other Po-Circo-like virus detected in China. Green marks the four strains of the Po-Circo-like virus found in the United States.

A novel CRESS DNA virus was first detected in pig intestinal tissue in the United States (Shan et al., 2011). The virus called the PCL virus, is similar to PCVs, as it has a circular genome. One difference is that the PCL virus does not express a typical capsid protein (Cap). At present, PCL virus has been reported in the United States and China. PCL virus infection was reported in pig farms in six provinces of China. In this study, we studied of the genomic characteristics of PCL virus in Hunan Province, China. According to the amino acid sequence analysis of Rep protein, the PCL virus strains in this study were closely related to 21 and 22 strains in the United States and other virus strains in China. Fewer amino acid mutations on the Rep protein of these viruses were observed. However, it is very interesting that Bo-Circo-like virus also has high identity with the strains in this study (Figure 2, 3; Supplementary Table 2). In summary, we detected PCL virus for the first time in Hunan Province, China, and sequenced the whole genomes of four strains. Once again demonstrated the high similarity between Bo-circo-like virus and PCL virus, further indicating that PCL virus possible transmitted to non-porcine hosts. Further studies based on more information of molecular epidemiology will help better understanding origin, evolution and transmission patterns of PCL virus in pigs in China.

## Data availability statement

The original contributions presented in the study are included in the article/Supplementary material, further inquiries can be directed to the corresponding authors.

## Ethics statement

The animal study was reviewed and approved by South China Agricultural University Experimental Animal Welfare Ethics Committee.

## Author contributions

JM and YS: conceived and designed the experiments. CJ, MZ, and XL: performed the experiments. CJ, MZ, and YW: sample collection. CJ and MZ: analyzed the data and contributed to the writing. All authors contributed to the article and approved the submitted version.

## Funding

This work was financially supported by the Guangdong Major Project of Basic and Applied Basic Research (no. 2020B0301030007) and Natural Science Foundation of Guangdong Province (nos. 2022A1515012473 and 2020A1515010295).

## Conflict of interest

The authors declare that the research was conducted in the absence of any commercial or financial relationships that could be construed as a potential conflict of interest.

## Publisher’s note

All claims expressed in this article are solely those of the authors and do not necessarily represent those of their affiliated organizations, or those of the publisher, the editors and the reviewers. Any product that may be evaluated in this article, or claim that may be made by its manufacturer, is not guaranteed or endorsed by the publisher.

## Footnotes

^1^ https://blast.ncbi.nlm.nih.gov/Blast.cgi

^2^ https://www.ncbi.nlm.nih.gov/orffinder/

^3^ http://mfold.rna.albany.edu/?q=mfold
