# Supplementary material for: Genetic characterization of four strains porcine circovirus-like viruses in pigs with diarrhea in Hunan Province of China
Source: Front Microbiol. 2023 Mar 1;14:1126707. doi: 10.3389/fmicb.2023.1126707 (PMC10014920; doi:10.3389/fmicb.2023.1126707)
Supplement: Supplementary file 1 [file Table_1.DOCX]

Supplementary Material

Genetic characterization of four strains porcine circovirus-like viruses in pigs with diarrhea in Hunan Province of China

ChiHai Ji ^a, b,^ ^c†^, Meng Zeng ^a, b, c†^, Yingfang Wei ^d^, Xiaocheng Lv^a, b, c^, Yuan Sun ^a, b, c^*, Jingyun Ma ^a, b, c^*

*** Correspondence:** Corresponding Author: E-mail: E-mail: majy2400@scau.edu.cn (Jingyun Ma), sunyuan@scau.edu.cn (Yuan Sun).

**Supplementary Tables**

TABLE 2 The sequence identity within the PCLV Rep protein gene at the nucleotide (upper right) and amino acid (lower left, boldface) levels calculated using ClustalW method implemented in MegAlign.

| Reference strains and this study strains | 1 | 2 | 3 | 4 | 5 | 6 | 7 | 8 | 9 | 10 | 11 | 12 | 13 | 14 | 15 | 16 | 17 | 18 | 19 | 20 | 21 | 22 | 23 | 24 | 25 | 26 | 27 | 28 | 29 | 30 | 31 | 32 | 33 | 34 | 35 | 36 | 37 | 38 | 39 | 40 | 41 | 42 | 43 | 44 | 45 | 46 |
| --- | --- | --- | --- | --- | --- | --- | --- | --- | --- | --- | --- | --- | --- | --- | --- | --- | --- | --- | --- | --- | --- | --- | --- | --- | --- | --- | --- | --- | --- | --- | --- | --- | --- | --- | --- | --- | --- | --- | --- | --- | --- | --- | --- | --- | --- | --- |
| 1 JX26-2020 MZ153221.1 |  | 99.9 | 99.8 | 99.8 | 99.4 | 98.0 | 86.3 | 94.1 | 92.1 | 87.4 | 90.8 | 97.7 | 97.4 | 97.9 | 40.4 | 40.3 | 88.6 | 91.0 | 91.1 | 93.1 | 95.4 | 90.4 | 90.6 | 99.8 | 99.8 | 99.7 | 99.5 | 99.4 | 91.1 | 93.1 | 97.3 | 97.2 | 96.6 | 99.7 | 99.9 | 90.4 | 99.6 | 99.8 | 99.7 | 99.6 | 99.7 | 99.6 | 99.8 | 99.8 | 91.7 | 94.0 |
| 2 JX27-2020 MZ153222.1 | **100** |  | 99.7 | 99.7 | 99.2 | 97.9 | 86.1 | 94.0 | 92.0 | 87.2 | 90.7 | 97.6 | 97.3 | 97.7 | 40.3 | 40.2 | 88.7 | 90.9 | 91.0 | 93.0 | 95.3 | 90.2 | 90.5 | 99.7 | 99.7 | 99.6 | 99.4 | 99.2 | 91.0 | 93.0 | 97.2 | 97.1 | 96.5 | 99.6 | 99.8 | 90.2 | 99.5 | 99.7 | 99.6 | 99.5 | 99.6 | 99.5 | 99.7 | 99.7 | 91.6 | 94.1 |
| 3 JX28-2020 MZ153223.1 | **99.7** | **99.7** |  | 99.6 | 99.4 | 97.7 | 86.3 | 94.1 | 92.1 | 87.4 | 90.8 | 97.5 | 97.2 | 97.6 | 40.7 | 40.6 | 88.6 | 91.0 | 91.1 | 93.1 | 95.4 | 90.4 | 90.6 | 99.6 | 99.6 | 99.5 | 99.5 | 99.4 | 91.1 | 93.1 | 97.1 | 97.0 | 96.4 | 99.7 | 99.7 | 90.2 | 99.4 | 99.6 | 99.5 | 99.4 | 99.5 | 99.4 | 99.6 | 99.6 | 91.7 | 93.8 |
| 4 JX29-2020 MZ153224.1 | **99.7** | **99.7** | **99.4** |  | 99.4 | 98.0 | 86.4 | 94.2 | 92.2 | 87.5 | 90.8 | 97.9 | 97.5 | 98.0 | 40.6 | 40.4 | 88.7 | 91.2 | 91.3 | 93.2 | 95.4 | 90.5 | 90.7 | 99.8 | 99.8 | 99.7 | 99.5 | 99.4 | 91.3 | 93.2 | 97.3 | 97.2 | 96.6 | 99.7 | 99.9 | 90.5 | 99.6 | 99.6 | 99.7 | 99.6 | 99.9 | 99.8 | 100 | 100.0 | 91.9 | 94.0 |
| 5JX30-2020 MZ153225.1 | **99.7** | **99.7** | **99.4** | **99.4** |  | 97.5 | 86.4 | 94.3 | 92.1 | 87.4 | 90.8 | 97.4 | 97.1 | 97.5 | 40.4 | 40.3 | 88.6 | 90.9 | 91.0 | 93.2 | 95.3 | 90.6 | 90.6 | 99.4 | 99.4 | 99.2 | 99.5 | 99.4 | 91.0 | 93.2 | 97.1 | 97.0 | 96.4 | 99.7 | 99.5 | 90.7 | 99.1 | 99.1 | 99.2 | 99.1 | 99.2 | 99.1 | 99.4 | 99.4 | 91.9 | 94.0 |
| 6 PCL-AH-23 MZ773067.1 | **99.0** | **99.0** | **98.7** | **98.7** | **98.7** |  | 86.4 | 93.9 | 91.2 | 87.6 | 91.2 | 97.4 | 97.1 | 97.5 | 40.3 | 40.2 | 89.0 | 91.2 | 91.3 | 92.6 | 95.7 | 90.5 | 90.8 | 98.0 | 98.0 | 97.9 | 97.6 | 97.5 | 91.3 | 92.6 | 97.6 | 97.5 | 96.2 | 97.9 | 98.1 | 90.5 | 97.7 | 97.7 | 97.9 | 97.7 | 97.9 | 97.7 | 98.0 | 98.0 | 91.2 | 93.7 |
| 7 PCL-AH-25 MZ773068.1 | **84.7** | **84.7** | **84.4** | **84.4** | **84.4** | **84.4** |  | 87.1 | 85.9 | 87.9 | 84.1 | 86.3 | 86.4 | 86.6 | 40.0 | 39.9 | 83.8 | 84.4 | 84.4 | 85.9 | 86.8 | 84.7 | 84.7 | 86.3 | 86.3 | 86.1 | 86.4 | 86.0 | 84.4 | 85.9 | 87.2 | 87.3 | 87.0 | 86.4 | 86.3 | 85.2 | 86.3 | 86.0 | 86.5 | 86.0 | 86.3 | 86.1 | 86.4 | 86.4 | 85.6 | 86.3 |
| 8 PCLV-HB-2021 MZ960935.1 | **98.1** | **98.1** | **97.7** | **97.7** | **97.7** | **99.0** | **84.7** |  | 94.0 | 87.5 | 89.7 | 94.6 | 94.3 | 94.7 | 40.3 | 40.2 | 90.2 | 90.0 | 90.1 | 95.8 | 94.7 | 89.7 | 89.2 | 94.1 | 94.1 | 94.0 | 94.0 | 93.9 | 90.1 | 95.8 | 95.1 | 95.0 | 94.3 | 94.2 | 94.2 | 89.8 | 93.9 | 93.9 | 94.2 | 93.9 | 94.1 | 94.0 | 94.2 | 94.2 | 94.4 | 93.4 |
| 9 21 JF713716.1 | **97.7** | **97.7** | **97.4** | **97.4** | **97.4** | **97.4** | **84.4** | **97.1** |  | 87.6 | 88.7 | 92.7 | 92.4 | 92.8 | 39.1 | 39.0 | 89.8 | 89.9 | 90.0 | 94.4 | 92.3 | 89.7 | 89.1 | 92.1 | 92.1 | 92.0 | 92.2 | 91.9 | 90.0 | 94.4 | 92.1 | 92.2 | 92.2 | 92.2 | 92.2 | 89.6 | 92.1 | 91.9 | 92.4 | 91.9 | 92.1 | 92.0 | 92.2 | 92.2 | 94.2 | 92.2 |
| 10 22 JF713717.1 | **90.3** | **90.3** | **90.0** | **90.0** | **90.0** | **91.0** | **86.6** | **91.3** | **90.3** |  | 85.7 | 88.1 | 88.2 | 88.4 | 40.1 | 40.0 | 84.7 | 86.5 | 86.7 | 86.2 | 86.8 | 86.8 | 86.5 | 87.4 | 87.4 | 87.2 | 87.5 | 87.1 | 86.7 | 86.2 | 88.6 | 88.7 | 88.0 | 87.5 | 87.4 | 86.5 | 87.5 | 87.1 | 87.6 | 87.4 | 87.4 | 87.2 | 87.5 | 87.5 | 86.7 | 87.1 |
| 11 Po-Circo-like virus HN14 | **97.4** | **97.4** | **97.1** | **97.1** | **97.1** | **97.7** | **85.0** | **96.8** | **95.8** | **90.3** |  | 91.2 | 91.1 | 91.3 | 39.6 | 39.4 | 86.7 | 94.4 | 94.5 | 89.4 | 90.6 | 94.2 | 94.2 | 90.8 | 90.8 | 90.7 | 90.7 | 90.6 | 94.5 | 89.4 | 91.3 | 91.2 | 91.0 | 90.9 | 90.9 | 90.5 | 90.8 | 90.6 | 90.9 | 90.8 | 90.7 | 90.6 | 90.8 | 90.8 | 89.0 | 91.2 |
| 12 Po-Circo-like virus HN39-01 | **99.0** | **99.0** | **98.7** | **98.7** | **98.7** | **99.4** | **84.7** | **98.4** | **97.4** | **90.3** | **97.7** |  | 99.7 | 99.7 | 40.7 | 40.6 | 89.2 | 91.3 | 91.4 | 93.5 | 95.3 | 90.6 | 90.7 | 97.7 | 97.7 | 97.6 | 97.4 | 97.3 | 91.4 | 93.5 | 96.8 | 96.7 | 96.5 | 97.6 | 97.9 | 90.6 | 97.7 | 97.5 | 97.9 | 97.5 | 97.7 | 97.6 | 97.9 | 97.9 | 91.6 | 94.1 |
| 13 Po-Circo-like virus HN39-02 | **99.0** | **99.0** | **98.7** | **98.7** | **98.7** | **99.4** | **84.7** | **98.4** | **97.4** | **90.3** | **97.7** | **100** |  | 99.4 | 40.8 | 40.7 | 88.9 | 91.0 | 91.1 | 93.1 | 95.0 | 90.5 | 90.6 | 97.4 | 97.4 | 97.3 | 97.1 | 97.0 | 91.1 | 93.1 | 96.5 | 96.4 | 96.1 | 97.3 | 97.5 | 90.2 | 97.4 | 97.2 | 97.5 | 97.2 | 97.4 | 97.3 | 97.5 | 97.5 | 91.3 | 93.8 |
| 14 Po-Circo-like virus HN75 | **99.0** | **99.0** | **98.7** | **98.7** | **98.7** | **99.4** | **84.7** | **98.4** | **97.4** | **90.3** | **97.7** | **100** | **100** |  | 40.8 | 40.7 | 89.3 | 91.4 | 91.5 | 93.8 | 95.2 | 90.9 | 91.0 | 97.9 | 97.9 | 97.7 | 97.5 | 97.4 | 91.5 | 93.8 | 96.9 | 96.8 | 96.6 | 97.7 | 98.0 | 90.7 | 97.9 | 97.6 | 98.0 | 97.6 | 97.9 | 97.7 | 98.0 | 98.0 | 92.0 | 94.4 |
| 15 21 strain 288-4 MW847276.1 | **96.8** | **96.8** | **96.5** | **96.5** | **96.5** | **96.8** | **85.3** | **97.1** | **96.5** | **91.6** | **97.1** | **96.8** | **96.8** | **96.8** |  | 99.7 | 39.0 | 40.2 | 40.2 | 39.7 | 40.7 | 39.6 | 40.2 | 40.4 | 40.6 | 40.4 | 40.4 | 40.3 | 40.2 | 39.7 | 40.6 | 40.6 | 39.9 | 40.6 | 40.6 | 39.2 | 40.6 | 40.4 | 40.6 | 40.4 | 40.4 | 40.3 | 40.6 | 40.6 | 40.2 | 39.6 |
| 16 21 strain 302-4 MW847277.1 | **96.5** | **96.5** | **96.1** | **96.1** | **96.1** | **96.5** | **85.7** | **96.8** | **96.1** | **91.9** | **96.8** | **96.5** | **96.5** | **96.5** | **99.7** |  | 38.9 | 40.1 | 40.1 | 39.6 | 40.6 | 39.4 | 40.3 | 40.3 | 40.4 | 40.3 | 40.3 | 40.2 | 40.1 | 39.6 | 40.4 | 40.4 | 39.8 | 40.4 | 40.5 | 39.1 | 40.4 | 40.3 | 40.4 | 40.3 | 40.3 | 40.2 | 40.4 | 40.4 | 40.1 | 39.4 |
| 17 Bo-Circo-like virus CH MH316857.1 | **93.2** | **93.2** | **92.8** | **92.8** | **93.2** | **93.2** | **85.0** | **92.8** | **92.5** | **87.3** | **93.2** | **93.2** | **93.2** | **93.2** | **92.8** | **93.2** |  | 87.7 | 87.8 | 89.5 | 88.7 | 88.1 | 87.4 | 88.7 | 88.6 | 88.5 | 88.5 | 88.4 | 87.8 | 89.5 | 89.1 | 89.0 | 88.1 | 88.7 | 88.7 | 87.8 | 88.6 | 88.4 | 88.7 | 88.4 | 88.6 | 88.5 | 88.7 | 88.7 | 89.7 | 89.2 |
| 18 CHZ09 MW881207.1 | **96.8** | **96.8** | **96.5** | **97.1** | **96.5** | **97.1** | **84.7** | **96.1** | **95.2** | **89.7** | **99.0** | **97.1** | **97.1** | **97.1** | **96.1** | **95.8** | **92.5** |  | 99.6 | 89.4 | 90.8 | 95.7 | 94.9 | 91.0 | 91.0 | 90.9 | 90.9 | 90.8 | 99.6 | 89.4 | 91.2 | 91.1 | 90.4 | 91.1 | 91.1 | 90.4 | 91.0 | 90.8 | 91.1 | 91.0 | 91.1 | 91.0 | 91.2 | 91.2 | 89.1 | 90.8 |
| 19 CMM06 MW881205.1 | **96.8** | **96.8** | **96.5** | **97.1** | **96.5** | **97.1** | **84.7** | **96.1** | **95.2** | **89.7** | **99.4** | **97.1** | **97.1** | **97.1** | **96.5** | **96.1** | **92.5** | **99.7** |  | 89.5 | 91.0 | 95.8 | 95.0 | 91.1 | 91.1 | 91.0 | 91.0 | 90.9 | 100 | 89.5 | 91.3 | 91.2 | 90.5 | 91.2 | 91.2 | 90.6 | 91.1 | 90.9 | 91.2 | 91.1 | 91.2 | 91.1 | 91.3 | 91.3 | 89.2 | 91.0 |
| 20 CQY09 MW881206.1 | **97.7** | **97.7** | **97.4** | **97.4** | **97.4** | **98.1** | **83.4** | **97.7** | **96.8** | **89.4** | **96.5** | **98.1** | **98.1** | **98.1** | **95.5** | **95.2** | **92.8** | **95.8** | **95.8** |  | 94.0 | 89.2 | 88.9 | 93.1 | 93.1 | 93.0 | 93.2 | 92.9 | 89.5 | 100 | 92.7 | 92.8 | 92.9 | 93.2 | 93.2 | 89.5 | 92.9 | 92.9 | 93.2 | 92.9 | 93.1 | 93.0 | 93.2 | 93.2 | 95.4 | 92.3 |
| 21 CSW10 QYM90074.1 | **97.7** | **97.7** | **97.4** | **97.4** | **97.4** | **98.7** | **84.4** | **97.7** | **96.8** | **90.6** | **96.5** | **98.1** | **98.1** | **98.1** | **95.5** | **95.8** | **92.5** | **95.8** | **95.8** | **97.4** |  | 89.9 | 90.0 | 95.4 | 95.4 | 95.3 | 95.5 | 95.2 | 91.0 | 94.0 | 95.5 | 95.6 | 95.4 | 95.5 | 95.5 | 89.6 | 95.2 | 95.2 | 95.3 | 95.2 | 95.3 | 95.2 | 95.4 | 95.4 | 92.5 | 92.0 |
| 22 CZH12 QYM90082.1 | **96.8** | **96.8** | **96.5** | **96.5** | **97.1** | **97.1** | **85.0** | **96.8** | **95.2** | **90.3** | **99.4** | **97.1** | **97.1** | **97.1** | **97.1** | **96.8** | **92.8** | **98.4** | **98.7** | **95.8** | **95.8** |  | 96.9 | 90.4 | 90.4 | 90.2 | 90.2 | 90.1 | 95.8 | 89.2 | 90.7 | 90.6 | 89.6 | 90.5 | 90.5 | 91.1 | 90.6 | 90.1 | 90.7 | 90.4 | 90.4 | 90.2 | 90.5 | 90.5 | 88.2 | 90.6 |
| 23 CZQ11 QYM90078.1 | **97.4** | **97.4** | **97.1** | **97.1** | **97.1** | **97.7** | **85.0** | **96.8** | **95.8** | **90.3** | **100** | **97.7** | **97.7** | **97.7** | **97.1** | **96.8** | **93.2** | **99.0** | **99.4** | **96.5** | **96.5** | **99.4** |  | 90.6 | 90.6 | 90.5 | 90.5 | 90.4 | 95.0 | 88.9 | 91.1 | 91.0 | 90.2 | 90.7 | 90.7 | 90.1 | 90.6 | 90.4 | 90.7 | 90.6 | 90.6 | 90.5 | 90.7 | 90.7 | 88.2 | 90.5 |
| 24 FJ9-2020 MZ191157.1 | **99.7** | **99.7** | **99.4** | **99.4** | **99.4** | **98.7** | **84.7** | **97.7** | **97.4** | **90.0** | **97.1** | **98.7** | **98.7** | **98.7** | **96.5** | **96.1** | **93.2** | **96.5** | **96.5** | **97.4** | **97.4** | **96.5** | **97.1** |  | 99.8 | 99.7 | 99.5 | 99.4 | 91.1 | 93.1 | 97.3 | 97.2 | 96.6 | 99.7 | 99.9 | 90.4 | 99.6 | 99.6 | 99.7 | 99.6 | 99.7 | 99.6 | 99.8 | 99.8 | 91.7 | 94.0 |
| 25 FJ10-2020 MZ191158.1 | **99.7** | **99.7** | **99.4** | **99.4** | **99.4** | **98.7** | **84.4** | **97.7** | **97.4** | **90.0** | **97.1** | **98.7** | **98.7** | **98.7** | **96.5** | **96.1** | **92.8** | **96.5** | **96.5** | **97.4** | **97.4** | **96.5** | **97.1** | **99.4** |  | 99.7 | 99.5 | 99.4 | 91.1 | 93.1 | 97.3 | 97.2 | 96.6 | 99.7 | 99.9 | 90.4 | 99.6 | 99.6 | 99.7 | 99.6 | 99.7 | 99.6 | 99.8 | 99.8 | 91.7 | 94.0 |
| 26 FJ11-2020 MZ191159.1 | **99.4** | **99.4** | **99.0** | **99.0** | **99.0** | **98.4** | **84.0** | **97.4** | **97.1** | **89.7** | **96.8** | **98.4** | **98.4** | **98.4** | **96.1** | **95.8** | **92.5** | **96.1** | **96.1** | **97.1** | **97.1** | **96.1** | **96.8** | **99.0** | **99.0** |  | 99.4 | 99.2 | 91.0 | 93.0 | 97.2 | 97.1 | 96.5 | 99.6 | 99.8 | 90.2 | 99.5 | 99.5 | 99.6 | 99.5 | 99.6 | 99.5 | 99.7 | 99.7 | 91.6 | 93.9 |
| 27 FJ12-2020 MZ191160.1 | **99.4** | **99.4** | **99.0** | **99.0** | **99.0** | **98.4** | **84.4** | **97.4** | **97.7** | **90.0** | **96.8** | **98.4** | **98.4** | **98.4** | **96.1** | **95.8** | **92.5** | **96.1** | **96.1** | **97.7** | **97.7** | **96.1** | **96.8** | **99.0** | **99.0** | **98.7** |  | 99.5 | 91.0 | 93.2 | 97.0 | 97.1 | 96.5 | 99.8 | 99.6 | 90.4 | 99.2 | 99.2 | 99.4 | 99.2 | 99.4 | 99.2 | 99.5 | 99.5 | 91.9 | 93.7 |
| 28 FJ13-2020 MZ191161.1 | **99.0** | **99.0** | **98.7** | **98.7** | **98.7** | **98.1** | **83.7** | **97.1** | **96.8** | **89.4** | **96.5** | **98.1** | **98.1** | **98.1** | **95.8** | **95.5** | **92.2** | **95.8** | **95.8** | **96.8** | **96.8** | **95.8** | **96.5** | **98.7** | **98.7** | **98.4** | **98.4** |  | 90.9 | 92.9 | 96.9 | 96.8 | 96.1 | 99.7 | 99.5 | 90.2 | 99.1 | 99.1 | 99.2 | 99.1 | 99.2 | 99.1 | 99.4 | 99.4 | 91.5 | 93.6 |
| 29 GD06 MW166350.1 | **96.8** | **96.8** | **96.5** | **97.1** | **96.5** | **97.1** | **84.7** | **96.1** | **95.2** | **89.7** | **99.4** | **97.1** | **97.1** | **97.1** | **96.5** | **96.1** | **92.5** | **99.7** | **100** | **95.8** | **95.8** | **98.7** | **99.4** | **96.5** | **96.5** | **96.1** | **96.1** | **95.8** |  | 89.5 | 91.3 | 91.2 | 90.5 | 91.2 | 91.2 | 90.6 | 91.1 | 90.9 | 91.2 | 91.1 | 91.2 | 91.1 | 91.3 | 91.3 | 89.2 | 91.0 |
| 30 GD09 MW166351.1 | **97.7** | **97.7** | **97.4** | **97.4** | **97.4** | **98.1** | **83.4** | **97.7** | **96.8** | **89.4** | **96.5** | **98.1** | **98.1** | **98.1** | **95.5** | **95.2** | **92.8** | **95.8** | **95.8** | **100** | **97.4** | **95.8** | **96.5** | **97.4** | **97.4** | **97.1** | **97.7** | **96.8** | **95.8** |  | 92.7 | 92.8 | 92.9 | 93.2 | 93.2 | 89.5 | 92.9 | 92.9 | 93.2 | 92.9 | 93.1 | 93.0 | 93.2 | 93.2 | 95.4 | 92.3 |
| 31 GX14 MN263296.1 | **98.4** | **98.4** | **98.1** | **98.1** | **98.1** | **99.4** | **84.4** | **99.0** | **97.4** | **91.0** | **97.1** | **98.7** | **98.7** | **98.7** | **96.8** | **96.5** | **93.2** | **96.5** | **96.5** | **97.4** | **98.1** | **96.5** | **97.1** | **98.1** | **98.1** | **97.7** | **97.7** | **97.4** | **96.5** | **97.4** |  | 99.9 | 96.2 | 97.2 | 97.4 | 91.3 | 97.1 | 97.1 | 97.4 | 97.1 | 97.2 | 97.1 | 97.3 | 97.3 | 92.7 | 94.9 |
| 32 GX15 MN263298.1 | **98.1** | **98.1** | **97.7** | **97.7** | **97.7** | **99.0** | **84.4** | **98.7** | **97.7** | **91.0** | **96.8** | **98.4** | **98.4** | **98.4** | **96.5** | **96.1** | **92.8** | **96.1** | **96.1** | **97.7** | **98.4** | **96.1** | **96.8** | **97.7** | **97.7** | **97.4** | **98.1** | **97.1** | **96.1** | **97.7** | **99.7** |  | 96.4 | 97.1 | 97.3 | 91.2 | 97.0 | 97.0 | 97.3 | 97.0 | 97.1 | 97.0 | 97.2 | 97.2 | 92.8 | 94.7 |
| 33 GX19 MN263297.1 | **97.7** | **97.7** | **97.4** | **97.4** | **97.4** | **98.1** | **85.3** | **97.7** | **97.4** | **91.0** | **96.5** | **98.7** | **98.7** | **98.7** | **96.8** | **96.5** | **92.2** | **95.8** | **95.8** | **97.4** | **97.4** | **96.5** | **96.5** | **97.4** | **97.4** | **97.1** | **97.7** | **96.8** | **95.8** | **97.4** | **97.4** | **97.7** |  | 96.5 | 96.7 | 90.1 | 96.4 | 96.4 | 96.5 | 96.6 | 96.5 | 96.4 | 96.6 | 96.6 | 91.9 | 93.7 |
| 34 JX13-2020 MZ191162.1 | **100** | **100** | **99.7** | **99.7** | **99.7** | **99.0** | **84.7** | **98.1** | **97.7** | **90.3** | **97.4** | **99.0** | **99.0** | **99.0** | **96.8** | **96.5** | **93.2** | **96.8** | **96.8** | **97.7** | **97.7** | **96.8** | **97.4** | **99.7** | **99.7** | **99.4** | **99.4** | **99.0** | **96.8** | **97.7** | **98.4** | **98.1** | **97.7** |  | 99.8 | 90.6 | 99.5 | 99.5 | 99.6 | 99.5 | 99.6 | 99.5 | 99.7 | 99.7 | 91.9 | 93.9 |
| 35 JX14-2020 MZ191163.1 | **100** | **100** | **99.7** | **99.7** | **99.7** | **99.0** | **84.7** | **98.1** | **97.7** | **90.3** | **97.4** | **99.0** | **99.0** | **99.0** | **96.8** | **96.5** | **93.2** | **96.8** | **96.8** | **97.7** | **97.7** | **96.8** | **97.4** | **99.7** | **99.7** | **99.4** | **99.4** | **99.0** | **96.8** | **97.7** | **98.4** | **98.1** | **97.7** | **100** |  | 90.5 | 99.7 | 99.7 | 99.8 | 99.7 | 99.8 | 99.7 | 99.9 | 99.9 | 91.8 | 94.1 |
| 36 JX15-2020 MZ191164.1 | **96.1** | **96.1** | **95.8** | **95.8** | **95.8** | **96.5** | **84.0** | **96.1** | **94.5** | **90.0** | **96.8** | **95.8** | **95.8** | **95.8** | **96.1** | **95.8** | **91.2** | **95.8** | **96.1** | **95.2** | **95.2** | **96.8** | **96.8** | **95.8** | **95.8** | **95.5** | **95.5** | **95.2** | **96.1** | **95.2** | **95.8** | **95.5** | **95.2** | **96.1** | **96.1** |  | 90.6 | 90.1 | 90.7 | 90.4 | 90.4 | 90.2 | 90.5 | 90.5 | 89.7 | 91.0 |
| 37 JX16-2020 MZ191165.1 | **99.7** | **99.7** | **99.4** | **99.4** | **99.4** | **98.7** | **84.4** | **97.7** | **97.4** | **90.0** | **97.1** | **98.7** | **98.7** | **98.7** | **96.5** | **96.1** | **92.8** | **96.5** | **96.5** | **97.4** | **97.4** | **96.5** | **97.1** | **99.4** | **99.4** | **99.0** | **99.0** | **98.7** | **96.5** | **97.4** | **98.1** | **97.7** | **97.4** | **99.7** | **99.7** | **95.8** |  | 99.4 | 99.7 | 99.4 | 99.5 | 99.4 | 99.6 | 99.6 | 91.5 | 93.8 |
| 38 JX17-2020 MZ191166.1 | **99.7** | **99.7** | **99.4** | **99.4** | **99.4** | **98.7** | **84.4** | **97.7** | **97.4** | **90.0** | **97.1** | **98.7** | **98.7** | **98.7** | **96.5** | **96.1** | **92.8** | **96.5** | **96.5** | **97.4** | **97.4** | **96.5** | **97.1** | **99.4** | **99.4** | **99.0** | **99.0** | **98.7** | **96.5** | **97.4** | **98.1** | **97.7** | **97.4** | **99.7** | **99.7** | **95.8** | **99.4** |  | 99.5 | 99.4 | 99.5 | 99.4 | 99.6 | 99.6 | 91.5 | 93.8 |
| 39 JX18-2020 MZ153226.1 | **100** | **100** | **99.7** | **99.7** | **99.7** | **99.0** | **84.7** | **98.1** | **97.7** | **90.3** | **97.4** | **99.0** | **99.0** | **99.0** | **96.8** | **96.5** | **93.2** | **96.8** | **96.8** | **97.7** | **97.7** | **96.8** | **97.4** | **99.7** | **99.7** | **99.4** | **99.4** | **99.0** | **96.8** | **97.7** | **98.4** | **98.1** | **97.7** | **100** | **100** | **96.1** | **99.7** | **99.7** |  | 99.5 | 99.6 | 99.5 | 99.7 | 99.7 | 91.9 | 94.1 |
| 40 JX19-2020 MZ153227.1 | **99.7** | **99.7** | **99.4** | **99.4** | **99.4** | **98.7** | **84.4** | **97.7** | **97.4** | **90.0** | **97.1** | **98.7** | **98.7** | **98.7** | **96.5** | **96.1** | **92.8** | **96.5** | **96.5** | **97.4** | **97.4** | **96.5** | **97.1** | **99.4** | **99.4** | **99.0** | **99.0** | **98.7** | **96.5** | **97.4** | **98.1** | **97.7** | **97.4** | **99.7** | **99.7** | **95.8** | **99.4** | **99.4** | **99.7** |  | 99.5 | 99.4 | 99.6 | 99.6 | 91.5 | 93.8 |
| 41 JX20-2020 MZ153228.1 | **99.4** | **99.4** | **99.0** | **99.7** | **99.0** | **98.4** | **84.0** | **97.4** | **97.1** | **89.7** | **96.8** | **98.4** | **98.4** | **98.4** | **96.1** | **95.8** | **92.5** | **96.8** | **96.8** | **97.1** | **97.1** | **96.1** | **96.8** | **99.0** | **99.0** | **98.7** | **98.7** | **98.4** | **96.8** | **97.1** | **97.7** | **97.4** | **97.1** | **99.4** | **99.4** | **95.5** | **99.0** | **99.0** | **99.4** | **99.0** |  | 99.9 | 99.9 | 99.9 | 91.7 | 93.9 |
| 42 JX21-2020 MZ153229.1 | **99.4** | **99.4** | **99.0** | **99.7** | **99.0** | **98.4** | **84.0** | **97.4** | **97.1** | **89.7** | **96.8** | **98.4** | **98.4** | **98.4** | **96.1** | **95.8** | **92.5** | **96.8** | **96.8** | **97.1** | **97.1** | **96.1** | **96.8** | **99.0** | **99.0** | **98.7** | **98.7** | **98.4** | **96.8** | **97.1** | **97.7** | **97.4** | **97.1** | **99.4** | **99.4** | **95.5** | **99.0** | **99.0** | **99.4** | **99.0** | **100** |  | 99.8 | 99.8 | 91.6 | 93.8 |
| 43 JX22-2020 MZ153230.1 | **99.7** | **99.7** | **99.4** | **100** | **99.4** | **98.7** | **84.4** | **97.7** | **97.4** | **90.0** | **97.1** | **98.7** | **98.7** | **98.7** | **96.5** | **96.1** | **92.8** | **97.1** | **97.1** | **97.4** | **97.4** | **96.5** | **97.1** | **99.4** | **99.4** | **99.0** | **99.0** | **98.7** | **97.1** | **97.4** | **98.1** | **97.7** | **97.4** | **99.7** | **99.7** | **95.8** | **99.4** | **99.4** | **99.7** | **99.4** | **99.7** | **99.7** |  | 100.0 | 91.9 | 94.0 |
| 44 JX23-2020 MZ153231.1 | **99.7** | **99.7** | **99.4** | **100** | **99.4** | **98.7** | **84.4** | **97.7** | **97.4** | **90.0** | **97.1** | **98.7** | **98.7** | **98.7** | **96.5** | **96.1** | **92.8** | **97.1** | **97.1** | **97.4** | **97.4** | **96.5** | **97.1** | **99.4** | **99.4** | **99.0** | **99.0** | **98.7** | **97.1** | **97.4** | **98.1** | **97.7** | **97.4** | **99.7** | **99.7** | **95.8** | **99.4** | **99.4** | **99.7** | **99.4** | **99.7** | **99.7** | **100** |  | 91.9 | 94.0 |
| 45 JX24-2020 MZ153232.1 | **96.1** | **96.1** | **95.8** | **95.8** | **95.8** | **96.5** | **82.7** | **96.8** | **96.5** | **89.4** | **94.2** | **95.8** | **95.8** | **95.8** | **94.8** | **94.5** | **91.9** | **93.5** | **93.5** | **97.1** | **95.8** | **93.5** | **94.2** | **95.8** | **95.8** | **95.5** | **96.1** | **95.2** | **93.5** | **97.1** | **96.5** | **96.8** | **95.8** | **96.1** | **96.1** | **94.2** | **95.8** | **95.8** | **96.1** | **95.8** | **95.5** | **95.5** | **95.8** | **95.8** |  | 91.9 |
| 46 JX25-2020 MZ153233.1 | **98.4** | **98.4** | **98.1** | **98.1** | **98.1** | **98.4** | **85.3** | **98.1** | **96.8** | **91.0** | **97.4** | **98.4** | **98.4** | **98.4** | **98.4** | **98.1** | **93.5** | **96.8** | **96.8** | **97.1** | **97.1** | **97.4** | **97.4** | **98.1** | **98.1** | **97.7** | **97.7** | **97.4** | **96.8** | **97.1** | **97.7** | **97.4** | **97.7** | **98.4** | **98.4** | **96.5** | **98.1** | **98.1** | **98.4** | **98.1** | **97.7** | **97.7** | **98.1** | **98.1** | **95.2** |  |
